# Adolf Beck: A pioneer in electroencephalography in between Richard Caton and Hans Berger

**DOI:** 10.2478/v10053-008-0148-3

**Published:** 2013-12-31

**Authors:** Anton Coenen, Oksana Zayachkivska

**Affiliations:** 1 Donders Centre for Cognition, Radboud University Nijmegen, The Netherlands; 2 Department of Physiology, Lviv National Medical University, Lviv, Ukraine

**Keywords:** Adolf Beck, pioneer in electroencephalography, spontaneous oscillations, evoked potentials, desynchronization of brain waves, Richard Caton, Hans Berger

## Abstract

Adolf Beck, born in 1863 in Kraków (Poland), joined the Department of Physiology of the Jagiellonian University in 1889, to work directly under the prominent professor in physiology Napoleon Cybulski. Following his suggestion, Beck started studies on the electrical brain activity of animals. He recorded negative electrical potentials in several brain areas evoked by peripheral sensory impulses. Using this technique, Beck localised various centres in the brain of several animal species. In doing this, he discovered continuous electrical oscillations in the electrical brain activity and noted that these oscillations ceased after sensory stimulation. This was the first description of desynchronization in electrical brain potentials. He published these findings in 1890 in the German *Centralblatt für Physiologie*. Immediately, an intense discussion arose under physiologists on the question who could claim being the founder of electroencephalography. Ultimately, Richard Caton from Liverpool showed that he had performed similar experiments in monkeys years earlier. Nevertheless, Beck added several new elements to the nature of electrical brain activity, such as evoked potentials and desynchronization. In looking back, Adolf Beck can be regarded, next to Richard Caton and together with Hans Berger (who later introduced the electrical brain recording method to humans), as one of the founders of electroencephalography.

## Adolf Beck: A pioneer in electroencephalography

**Figure 1.**
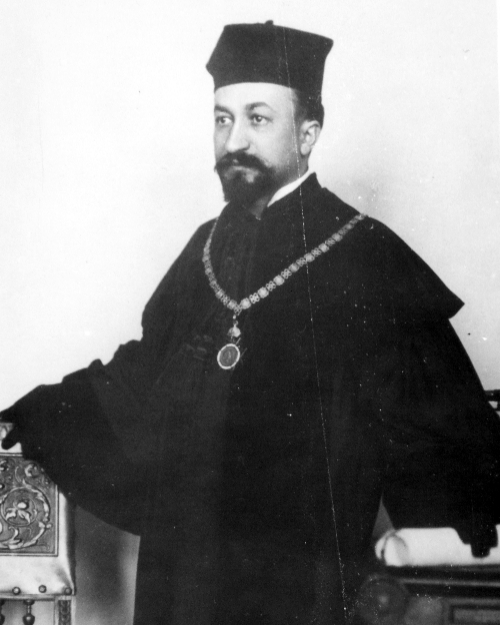
Adolf Beck on a photo taken in 1905, as Dean of the Medical Fa- culty of the University of Lemberg. In 1895 Beck moved from the Jagiellonian University in Kraków where he performed his electroencephalographic work to Lemberg/Lwów where he got a chair in physiology.

On the first of January 1863, Adolf Abraham Beck (Figure 1) was born to a family of a Jewish baker in Kraków, a city in Polish Galicia, the Austrian sector of partitioned Poland. After his study in medicine, in 1889 Beck began to work as an assistant of the famous physiologist Napoleon Nikodem Cybulski (1854-1919). Inspired by Du Bois-Reymond’s (1848) book *Untersuchungen über die thierische Elektrizität* [*Investigations on Animal Electricity*], Beck’s interest was directed towards the electrophysiology of the nervous system, in particular, the electrical response of a nerve evoked by sensory stimulation. This idea was suggested to him by Cybulski, since a lively debated question was whether it is possible to excite a nerve at all points of its pathway. Based on the fact that the activity is easily travelling along the nerve, the German Eduard Pflüger (1859) proposed that the neural excitation gathers strength in its passage down the nerve. This “avalanche theory” states that conduction depends not simply upon a wave-like pattern propagating, but upon a gradual increment of the potential energy in the nerve along its entire length. A student of Pflüger, the Russian Bronislav Verigo (1860-1925) was already engaged in research on the excitability of nerves, testing the idea that electrical activity is one of the forces of brain activity (Verigo, 1891).

## Beck’s Recordings of Electrical Brain Activity

Beck began to measure the excitability at two points of the spinal cord of the frog following the stimulation of the sciatic nerve. One electrode was placed at the lower spinal cord and the other at the entry of the spinal cord to the brain at the height of the cerebral hemispheres. It appeared that the electronegative variations following stimulation substantially changed at the level of the chord’s entry point into the brain. This was explained by the observation that the spontaneous current was already fluctuating, while the evoked activity added to these oscillations. Beck published his first findings in a Polish scientific journal in 1888 (Beck, 1888). A year later, Beck became graduate student at the Department of Physiology of the Jagiellonian University in Kraków where he began his extensive research on the electrical processes of the brain, the main topic of his doctorate work. The oscillations which Beck and Cybulski saw in the fluctuating baseline led these authors to the idea of continuously recording the spontaneous electrical brain activity. Beck finished his medical doctorate work with a cum laude graduation in 1890. The dissertation written in the Polish language appeared in 1891 (Beck, 1891). On the initiative of Mary Brazier, an expert on the history of neuroscience, this thesis was later translated into English (Beck, 1973).

**Figure 2.**
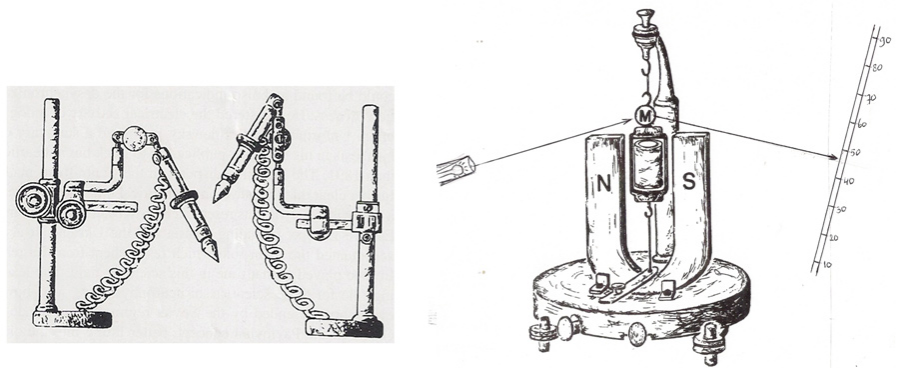
Left: the Du Bois-Reymond electrodes used by Beck for recording the electrical brain activity. These non-polarisable electrodes were made of cotton threads embedded in clay. Electrodes were connected to a D’Arsonval galvanometer which was provided with a tiny mirror (M), reflecting a light beam on a read-out scale. Recording cameras were not available.

One year earlier, Beck sent a summary of his extensive research in the form of a short manuscript entitled “Die Bestimmung der Localisation der Gehirn- und Rückenmarksfunctionen vermittelst der electrischen Erscheinungen” [“The Determination of the Localisation of the Brain and Spinal Cord Functions by Way of Electrical Appearances”] to the leading European physiology magazine *Centralblatt für Physiologie* (Beck, 1890). This paper became a classic in electrophysiology. In that brief article, Beck described his findings on the nature of electrical brain activity. He described the localisation of sensory modalities on the surface of cerebral cortex by electrical and sensory stimulation and by recording the electrical activities with clay electrodes and with a string galvanometer (Figure 2). In frogs as well as in paralysed dogs and rabbits, he explored the parts of the cortex which reacted upon stimulation with electronegativity. Beck did this for several sensory modalities. In doing this, Beck also found the spontaneous oscillations of brain potentials and showed that these fluctuations were not related to heart and breathing rhythms. Moreover, Beck mentioned the change in the potentials upon sensory stimulation. The evoked potentials were followed by a cessation of the fluctuations of the electrical waves as a consequence of afferent stimulation, either by electrical stimulation of the nervus ischiadicus or by peripheral stimulation with light flashes or handclaps. This brief paper attracted a lot of attention and can be considered as the first to describe “evoked potentials” as well as the desynchronisation in the electroencephalogram following stimulation (Coenen, Zayachkivska, & Bilski, 1998). It was clear that the then 27-year old Beck claimed to be the discoverer of the electrical brain activity that is nowadays known as the electroencephalogram.

## Claims on the Discovery of Electrical Brain Activity

**Figure 3.**
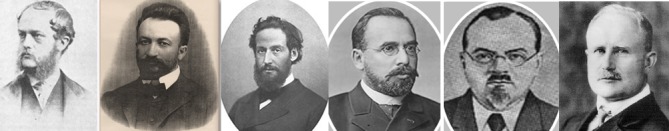
Pioneers in studying electrical brain activity. From left to right: Richard Caton (1842-1926) from Liverpool, Adolf Beck (1863-1942) from Kraków, Ernst Fleischl von Marxow (1846-1891) from Vienna, Vasili Yakovlevich Danilevsky (1852-1939) from Charkov, Vladimir Právdicz-Neminski (1879-1952) from Kiev, and Hans Berger (1873-1941) from Jena.

In the scientific domain of physiology, the paper of Beck evoked a polemic. A spate of claims for priority in discovering electrical brain activity followed. The first was from Ernst Fleischl von Marxow (1890), a prominent physiology professor at the University of Vienna (Figure 3). He wrote that he had already 7 years earlier deposited a sealed letter at the Imperial Academy of Sciences in Vienna containing claims on the discovery of electrical brain activity. Indeed in that letter indications of electrical brain activity were given but Fleischl’s observations missed crucial points. Fleischl who suffered from chronic pains and was treated by his friend Sigmund Freud with cocaine, was heavily addicted and died shortly after his letter in 1891. A second response to Beck’s paper came from Francis Gotch and Victor Horsley (Gotch & Horsley, 1891). Although they referred to papers slightly related to the subject, it was of interest that they mentioned the electrical response to sensory stimulation. Gotch, a direct colleague of Caton and the descriptor of the refractory phase that takes place between nerve impulses, performed experiments on the electrical responses of the mammalian spinal cord to cortical stimulation. He did this together with his brother in law, the famous Horsley, the designer of the stereotactic apparatus for brain research. Just as Fleischl, however, Gotch and Horsley overlooked essential elements, such as the spontaneous oscillations and the cessation of these fluctuations after stimulation.

The most interesting response, however, came from Vasili Y. Danilevsky (1891; see Figure 3), a scientist working at the University of Charkow (Russia, now Ukraine). Danilevsky studied at the University of Kazan in Russia together with Vladimir Ulyanov, later known as Lenin, and he finished his study at the University of Charkow in 1877. His doctoral thesis dating from that year was entitled *Investigations in the Physiology of the Brain* and was written in Russian. In his letter to the Centralblatt, Danilevsky mentioned his non-published doctoral thesis. Indeed in this manuscript a description of the spontaneously fluctuating brain potentials of a dog’s brain can be found and also indications for the changes in brain potentials after stimulation. Unfortunately, Danilevsky published a summary of his thesis in his response to Beck not earlier than 1891. Nevertheless, what results of the claims to the Centralblatt is that it is almost certain that Danilevsky was the first scientist after Caton who observed electrical potentials of the brain.

## Caton’s Decisive Claim

The discussion concerning the claim on the discovery of the electrical brain activity was abruptly ended by a letter of Richard Caton (1891) from the School of Medicine in Liverpool in England (Figure 3). Caton, a young medical physiologist, referred to a brief abstract of 10 sentences which he had published 15 years earlier in 1875 (Caton, 1875). In this abstract entitled *The Electric Currents of the Brain*, which appeared in the *British Medical Journal*, Caton described the spontaneous waxing and waning of the electrical activity recorded from the brain of rabbits and monkeys. The abstract appeared on the occasion of a meeting of the British Medical Association in 1875. Two years later in a longer paper published in the same journal, Caton (1877) more extensively described identical experiments with a larger number of animals, almost with the same results. Regrettable for Caton was that his findings “produced no single ripple in the pool of physiologists.” But it is now generally accepted that his short note from 1875 contains the first description of the electroencephalogram.

Richard Caton was an Edinburgh graduate in 1867. A year later he settled in Liverpool and became lecturer in physiology at the Royal Infirmary School of Medicine in Liverpool. Caton started with work on the animal brain, using non-polarisable electrodes and a string galvanometer. After defending his priority in having made the discovery of the electrical brain waves, he did no further work on the brain. For many years his family and colleagues were unaware of his discovery. This was possible partly because of many other things that he did in his life, but also because he took deliberate steps to hide the fact that he had worked on the brain of animals. The most important of these other activities was a study of the treatment of rheumatic heart disease. Caton’s interest in his university never waned, and he reached the high office of pro-chancellor. Later, he became a city councillor and devoted much time to the promotion of public health. In 1907 he was elected Lord Mayor of Liverpool and as such he became more well-known than as a brain scientist. Nonetheless Richard Caton is presently recognised as the discoverer of the electrical brain activity, which forms the basis of electroencephalography (Brazier, 1959, 1988).

## Human Brain Recordings of Berger

Almost 40 years after Beck, in 1929, Hans Berger published his first paper about recordings of electrical activity from the surface of the human brain (Berger, 1929; Figure 3). Berger was interested in clinical applications of electrical brain activity and especially in the relation of brain activity with “psychic energy.” He had a more sensitive double-coil galvanometer than his predecessors, while at the same time powerful amplifiers came on the scene. Already in 1924 he was able to record the electrical intra-cerebral brain activity in patients with skull defects but it lasted until 1927 before he could make recordings directly from the skull. His children, especially son Klaus and daughter Ilse, were main, obedient, but also often unwilling subjects. Berger was the first to record the electrical activity from an intact human skull and so promoted a non-invasive technique. Berger came to the conclusion that the discovery of the EEG was not only a major breakthrough in neurophysiology but also that this technology was of outstanding importance for its diagnostic value (Berger, 1929, 1969).

Born in 1873 in the town of Neuses, near Coburg, in the south of Germany, Berger got a doctorate of medicine at the University of Jena in 1897. In 1901 he became staff member at the Psychiatric Clinic of this university and Chairman in 1919. The important findings of Berger were largely ignored and neglected by the scientific community but slowly his international reputation was growing. This brought the modest Berger to the International Congress of Psychology in Paris in 1938 where he was almost recognised as a celebrity. Back in Germany he found, however, only humiliation especially by the Nazi regime which distrusted his work. The Nazis also forced him to give up his Chair at the Psychiatry Clinic in 1938 and closed down his laboratory. They even did not allow him to receive the Nobel Prize in Stockholm for which he was nominated. Berger fell in a severe and long depression and on the 1st of June 1941 he took his own life. Berger’s wife Freiin Ursula von Bülow had a hard time since son Klaus fell on the battlefield in Russia half a year later. Berger was the first to non-invasively record electrical activity of the human brain and so promoted the transfer of the brain recording technique from animals to humans.

A main implication was that Berger got the honour to be generally regarded as the grandfather of electroencephalography. In the history of electroencephalography, interest must also be directed to its two earlier discoverers: Richard Caton with his first note of 1875, and Adolf Beck with his 1890 paper. In looking back, it seems best to attribute the discovery of electroencephalography to the trio: to Richard Caton for his brief description of brain waves, to Adolf Beck for his extensive brain work in animals, and to Hans Berger for making the recording technique applicable to humans.

## Beck Appointed Professor in Lemberg

It is noteworthy to mention that the scientific activity of Beck was not limited to electrophysiology. Beck’s scientific interests were broad, and he extended his research to other fields, such as to general and visceral physiology. In 1894 Beck got his *venia legendi* (“habilitation”) in physiology with a thesis entitled *On Variations in Venous Pressure* (Beck, 1895). In May 1895, at the age of 32 Beck accepted the offer to be appointed as professor in physiology at the University of Lemberg. Lemberg was the capital of Galicia. In 1919 it came under Polish rule with the name Lwów, and in 1945 to the Soviet Union, renamed to *Lviv*. Nowadays, Lviv is a city in the west of Ukraine. Beck worked at the University of Lemberg/Lwów till 1935 and spent the rest of his life in this city. Beck started to build up a new Department of Physiology at the Medical Faculty and organised a modern electrophysiological laboratory. Besides his main focus on electrophysiology, together with his close colleague, Gustav Bikeles, Beck performed valuable research on many other neuroscientific and physiological topics, such as pain sensation, the cerebellum, and retinal receptors. In 1910 Beck took part in the organisation of the International Congress of Physiology in Vienna where he again closely collaborated with Napoleon Cybulski. With him and with Kraków colleague Sabina Jeleska-Macieszyna he continued joint electrophysiological work. The group was able to publish one of the first photographs of electroencephalographic potentials (Cybulski & Jeleska-Macieszyna, 1914).

The first registration of spontaneous electrical brain activity was made in 1913 by Wladimir Práwdicz-Neminski who worked at the Kiev University of St. Vladimir and later at the Ukrainian Academy of Sciences (Práwdicz-Neminski, 1913; see Figure 3). This researcher identified also the two patterns of rhythms in the electrical activity of dogs, initially denoted as *waves of the first order and waves of the second order*, later called *A-waves* and *B-waves*, and now known as the *alpha*- and *beta-waves*. He also coined the German term *Elektrocerebrogramm* for the electrical brain activity (Práwdicz-Neminski, 1925) which Berger (1929) changed to *elektrenkephalogramm*. In English this term was translated as *electroencephalogram*, abbreviated as EEG.

All activities of Beck were abruptly interrupted in 1914 with the outbreak of the First World War. Lemberg was occupied by the Russians. Beck was arrested by the Russian army on the 19th of June 1915, and in 1916 he was released by the intervention of the famous Russian scientist and Nobel Prize winner Ivan Pavlov (1849-1935), working in St. Petersburg, and a friend of Napoleon Cybulski. After several months Beck was able to return to Lemberg. In 1935 he authored a memoir on the adverse days of the First World War which became a genuine documentary chronicle of the events that took place at the university (Beck, 1935). In the year 1919 Beck lost his friend Napoleon Cybulski. Beck paid tribute to his old teacher as a scientist, a colleague, a friend of the university, and a great human being (Beck, 1919). Beck retired in 1930 at the age of 67 with the title *Professor honoris causa*, one of his many distinctions. He was a member of the Academy of Arts and Sciences in Kraków, of the Academy of Medical Science in Warsaw, as well of the Polish Academy of Sciences. Beck produced 180 publications and was nominated three times for the Nobel Prize in physiology, once in 1905, then in 1908, and finally in 1911, but he never received this high honour (Zayachkivska, Gzegotsky, & Coenen, 2012).

## Beck’s Tragedy in the Second World War

During the Second World War life became even more troubled and dangerous for Beck than during the First World War. Lwów was then occupied by the Nazis, and Beck, who was of Jewish origin, remained in the town and suffered many humiliations. Instead of hiding, he decided to stay in his house in the shadow of the university to which he had devoted so many years of his working life. But it became too risky, since many Jews were already murdered in the Janowska and Beec concentration camps, and several Lwów professors were killed on the Wuleckie Hills. The physiologist and medical doctor Zdzisaw Bieliski, the successor of Beck at the Department of Physiology, started to take care of his old teacher. Together with Beck’s son Henryk he found a hiding place for him. Just before his 80th birthday, Beck became unwell and Henryk and Bieliski brought him to the hospital. However, Beck was betrayed and at the last moment Henryk could hand his father a capsule with cyanide to enable him to commit suicide before the Nazis could send him to the gas chambers. This happened in August 1942 but the precise date is not known.

**Figure 4.**
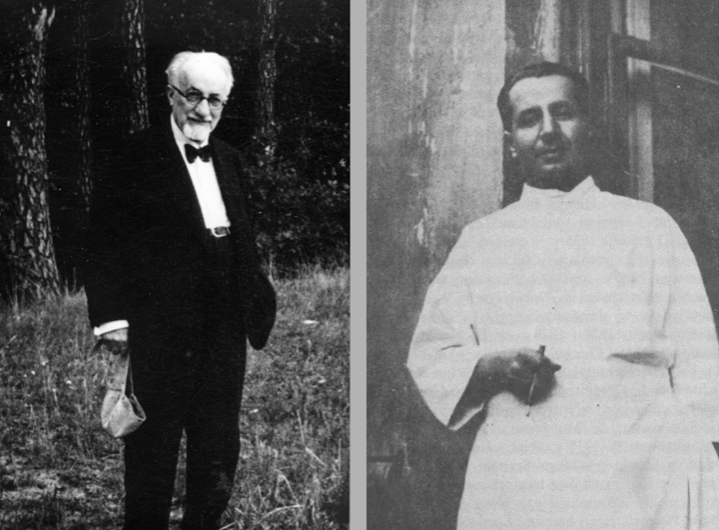
Left: One of the last photographs of Beck made in 1938. Right: Beck’s son Henryk. The outbreak of the Second World War is approaching, with all the dramas for the Beck family.

Given the tragedies in the Beck family, Beck’s suicide and the execution in Palmiry of Kazimierz Zakrzewski, the husband of Beck’s daughter Jadwiga, it was not surprising that Beck’s son Henryk, born in 1896, a medical doctor specialised in gynaecology and a unique artistic talented person, joined the Warsaw Uprising in 1944 (Figure 4). Henryk Beck with a military background in the Polish army became one of the leaders of the Uprising. After the final capitulation in 1944, he hid in the ruins of Warsaw and became one of the Robinson Crusoes of Warsaw. The life in the cellars, in suffocating air, heat, and darkness, while continuously being under extreme danger, was a genuine torture. Yet, Henryk Beck provided medical assistance to the wounded and, moreover, with his artistic talents he could manage to create a series of documentary drawings and watercolours about the war, his life, and his family under these conditions (Jaworska, 1982). Henryk Beck survived the occupation and in 1946 he got a chair in obstetrics in Wrocaw. Unfortunately, after some months he died of heart failure, undoubtedly following his sufferings of the war time. Beck’s daughter Jadwiga Beck-Zakrzewska was the only member of the Beck family who survived. In a moving obituary (Beck-Zakrzewska, 1973), she remembered her father Adolf Beck as a great scientist and humanist.
